# Cardiotoxic and Cardioprotective Effects of Methylene Blue in the Animal Model of Cardiac Ischemia and Reperfusion

**DOI:** 10.3390/biomedicines12112575

**Published:** 2024-11-11

**Authors:** Hezio Jadir Fernandes Junior, Erisvaldo Amarante de Araújo, José Antônio Machado Junior, Fabio Marinho Lutz Motta, Gabriela Ferrazzano Guarize, Lucas Chen Cheng, Junaid Tantray, Jand Venes Rolim Medeiros, Lucas Antonio Duarte Nicolau, Adriano Henrique Pereira Barbosa, Adriano Caixeta, Isadora S. Rocco, Solange Guizilini, Marcelo Pires-Oliveira, Murched Omar Taha, Afonso Caricati-Neto, Walter José Gomes, Fernando Sabia Tallo, Francisco Sandro Menezes-Rodrigues

**Affiliations:** 1 Postgraduate Program in Cardiology, Universidade Federal de São Paulo (UNIFESP), São Paulo 04024-002, SP, Brazil; heziojfj1@hotmail.com (H.J.F.J.); amarante.araujo@unifesp.br (E.A.d.A.); barbosa-ah@uol.com.br (A.H.P.B.); adriano.caixeta@unifesp.br (A.C.); isadora.rocco@unifesp.br (I.S.R.); sguizilini@unifesp.br (S.G.); wjgomes1012@gmail.com (W.J.G.); 2 Postgraduate Program in Interdisciplinary Surgical Science, Universidade Federal de São Paulo (UNIFESP), São Paulo 04024-002, SP, Brazil; ortopediamachado@gmail.com (J.A.M.J.); taha@uol.com.br (M.O.T.); 3 School of Medicine, Universidade Santo Amaro (UNISA), São Paulo 04829-300, SP, Brazil; fabiolutzmotta@gmail.com (F.M.L.M.); gabyfguarize@hotmail.com (G.F.G.); lucaschen98@hotmail.com (L.C.C.); 4 Department of Pharmacy, NIMS University, Jaipur 303121, Rajasthan, India; junaidtantray22@gmail.com; 5 Department of Biotechnology, Universidade Federal do Delta do Parnaíba (UFDPar), Parnaíba 64202-020, PI, Brazil; jandvenes@ufpi.edu.br (J.V.R.M.); lucasnicolau@ufpi.edu.br (L.A.D.N.); 6 Cardiovascular Surgery Discipline, Escola Paulista de Medicina, Universidade Federal de São Paulo (UNIFESP), São Paulo 04024-002, SP, Brazil; 7 School of Medicine, Centro Universitário UNIME, Lauro de Freitas 42702-420, BA, Brazil; marpoliv@umich.edu; 8 Department of Pharmacology, Escola Paulista de Medicina, Universidade Federal de São Paulo (UNIFESP), São Paulo 04023-062, SP, Brazil; caricatineto@gmail.com

**Keywords:** cardiac ischemia and reperfusion, myocardial injuries, cardiotoxicity, cardioprotection, arrhythmias, methylene blue

## Abstract

**Background/Objectives:** Treatment of patients with myocardial ischemic diseases crucially involves cardiac reperfusion (CR). However, oxidative stress and tissue lesions caused by CR may also lead to lethal complications, such as arrythmias and vasoplegic syndrome (VS). Although methylene blue (MB) has long been used to treat VS due to cardiac ischemia and reperfusion (CIR) and/or surgery because of its vascular effects, MB’s effects on the heart are unclear. Therefore, we investigated the potential cardioprotective or arrhythmogenic effects of MB in an animal model of CIR. To this end, 12–16-week-old male Wistar rats were divided into four experimental groups: (a) rats subjected to SHAM surgery with no ischemia; (b) rats subjected to CIR and treated with a vehicle (SS + CIR); and (c) rats subjected to CIR and treated with 2 mg/kg i.v. MB before ischemia (MB + ISQ) or (d) after ischemia but before reperfusion (ISQ + MB). An ECG analysis was used to evaluate the incidence of ventricular arrhythmias (VAs), atrioventricular blocks (AVBs), and lethality (LET) resulting from CIR. After CIR, rat hearts were removed for histopathological analysis and lipid hydroperoxide (LH) measurements. **Results:** The incidence of VA, AVB, and LET was significantly increased in the MB + ISQ group (VA = 100%; AVB = 100%; LET = 100%) but significantly reduced in the ISQ + MB group (VA = 42.8%; AVB = 28.5%; LET = 21.4%) compared with the SS + CIR group (VA = 85.7%; AVB = 71.4%; LET = 64.2%). LH concentration was significantly reduced in both MB-treated groups, but myocardial injuries were increased only in the MB + ISQ group when compared with the SS + CIR group. **Conclusions:** These results indicate that MB produces a biphasic effect on CIR, with cardiotoxic effects when administered before cardiac ischemia and cardioprotective effects when administered after ischemia but before cardiac reperfusion.

## 1. Introduction

Myocardial ischemia, such as that caused by acute myocardial infarctions, is a leading cause of damage to the structure and function of the heart, leading to severe clinical consequences. Tissue reperfusion is a crucial strategy for the prevention of ischemia-induced damage, and interventions include pharmacological and mechanical thrombolysis, coronary bypass surgery, etc. However, the most hazardous effector phase of cardiac ischemia and reperfusion (CIR) occurs when previously ischemic tissue suffers cardiac reperfusion; reperfusion injury, stabbing, and microvascular damage are the main causes of cell death, and they frequently worsen cardiac dysfunction and myocardial stunning. Additionally, CIR can also lead to heart rhythm abnormalities, particularly ventricular arrhythmias (VAs), including ventricular tachycardia, ventricular fibrillation, and premature ventricular contractions; Torsades de Pointes; and atrioventricular block (AVB) of the first, second, and third degree. These arrhythmic events, in turn, lead to sudden cardiac lethality (LET) [1,2,3].

These reperfusion injuries are predominantly triggered by an intense inflammatory process and an increase in pro-inflammatory cytokines such as interleukins-1 and -6 and tumoral necrosis factor-α, as well as oxidative stress resulting mainly from the metabolism of NO [4,5,6,7]. Inflammatory cytokines increase catecholamine secretion by the adrenal glands, which increases vasoconstriction and blood pressure; however, this hypersecretion subsequently leads to catecholamine depletion. Additionally, there is also a desensitization of α_1_-adrenoceptors present in blood vessels, causing an intense and continuous relaxation of vascular smooth muscle. This excessive vasodilation, in which the effect of NO predominates with no commensurate sympathetic counterpart, is called vasoplegic syndrome (VS) [4,5,6,7,8].

VS is a deadly complication consecutive to CIR and/or cardiovascular surgical procedures [7,8]. VS is characterized by hypotension, leading to the hypoperfusion of different and relevant organs, such as the brain, kidneys, and the heart itself. It is also characterized by normal or elevated cardiac output, low systemic vascular resistance, and decreased filling pressures, leading to a heightened rate of morbimortality [4,5,6,7,8]. The main therapeutic intervention to increase systemic vascular resistance and reduce vasopressor requirements in human VS is methylene blue (MB) [8,9,10].

MB was first used as a textile dye but has been employed in laboratories and clinics since the 1890s. By inhibiting the soluble guanylate cyclase (GC) [11,12,13] through the oxidation of its active heme core or the inactivation of its heme-deficient apoenzyme [11,12], MB counteracts NO-mediated vasodilatation. Furthermore, MB binds to and inactivates NO in addition to being a strong inhibitor of NOS [10]. Moreover, MB is useful in reducing ischemia-reperfusion syndrome [13]. It accomplishes this by competing with molecular oxygen for the transfer of electrons by xanthine oxidase, thereby inhibiting the production of free oxygen radicals and superoxide (reactive oxygen species) [14]. Because free-radical-mediated injury has been hypothesized to be one of the primary causes of post-ischemic reperfusion injury, MB, a type of free-radical scavenger, may provide an alternative treatment in post-resuscitation syndrome [4,5].

Generally, the beneficial effects of MB in VS are attributed to a reduction in vascular nitric oxide (NO) bioavailability, which basically results from its selective inhibition of GC and consequent inhibition of cellular responses mediated by cyclic guanosine monophosphate (cGMP) [6,7]. Nevertheless, in some animal models of CIR, MB also promotes the stabilization of systemic circulation without significantly increasing total peripheral resistance during reperfusion, instead decreasing lipid peroxidation and inflammation and reducing anoxic tissue injury in the heart [2,4,5]. MB also attenuates the elevation of plasma creatine kinase induced by CIR [15], suggesting that MB produces cardioprotective effects, as it does in different animal models of ischemia and reperfusion injury, including isolated rat lungs [16], lung transplantation [17], pig-controlled cardiopulmonary resuscitation [18], and graft reperfusion in orthotropic liver transplantation [19].

Despite the crucial role of MB in vasoplegic syndromes, we have previously not observed cardioprotection with MB in an animal model of CIR. On the contrary, when MB was administered before CIR, it had significant cardiotoxic effects that resulted in an increase in lethal arrhythmias [8]. The putative cardioprotective effects of MB are likely related to its attenuation of lesions associated with oxidative stress, which are more pronounced during reperfusion. Indeed, we have previously seen that the timing of interventions, such as ischemic preconditioning, is relevant for cardioprotection [20]. Thus, we compared the effect of MB when administered before ischemia or after ischemia but before reperfusion on myocardial structure, lipid peroxidation, electrical activity, and LET in rats subjected to CIR.

The present study shows that the incidence of AVB, VA, LET, myocardial injuries, and lipid hydroperoxide (LH) concentration related to CIR were significantly higher in the pre-ischemia MB group compared with non-treated CIR animals, suggesting that MB produced cardiotoxic effects when administered before ischemia. In contrast, the same parameters were significantly lower in the post-ischemia, before reperfusion MB group, suggesting that MB has useful cardioprotective effects when administered in this time window. These findings represent an important advancement in the understanding of the cardiac effects of MB and its safe use in the appropriate phase of lesions associated with cardiac ischemia and/or surgery.

## 2. Materials and Methods

### 2.1. Animals

Male adult (12–16-week-old) Wistar rats were provided by the Center for the Development of Experimental Models in Medicine and Biology (CEDEME) of the Escola Paulista de Medicina/Universidade Federal de São Paulo (UNIFESP). Rats were kept under standard conditions of nutrition, hydration, temperature, humidity, and luminosity until the moment of experimentation. All experimental procedures were approved by the Ethics Committee on Animal Use of UNIFESP (#9447210317) and were in accordance with the National Council for the Control of Animal Experimentation regulations (CONCEA, Brazil). All animal handling and surgical procedures were strictly conducted according to the NIH’s *Guide for the Care and Use of Laboratory Animals* (8th ed.).

### 2.2. Surgical Protocol for Induction of Cardiac Ischemia and Reperfusion (CIR)

Surgical procedures to induce CIR were performed in accordance with the methodology previously described by our research group [21,22]. In this CIR protocol, rats were anesthetized with 100 mg/kg ketamine and 10 mg/kg xylazine and mechanically ventilated [Harvard Apparatus, Boston]. After a 15 min stabilization period, the heart was exposed by left thoracotomy for mechanical occlusion of the left anterior descending artery with a plastic rod (ischemia). After 10 min of ischemia, the rod was removed to allow coronary reperfusion for 75 min. During all CIR protocols, cardiac electrical activity was monitored by an electrocardiogram (ECG) analysis using AqDados 7.02 software, while raw data were evaluated with AqDAnalysis 7 software [Lynx Tecnologia Ltd.a, São Paulo, Brazil] to record the incidences of ventricular arrhythmias (VAs), atrioventricular block (AVB), and lethality (LET) induced by CIR.

A total of 52 rats were randomly allocated into four experimental groups:

**(1) SHAM group (*n* = 10):** rats subjected to all surgical procedures performed in the CIR group, with the exception of garroting of the left anterior descending coronary artery;

**(2) SS + CIR group (*n* = 14):** rats subjected to the CIR protocol and treated with 0.9% saline solution (SS) intravenously (i.v.) administered through the left femoral vein before ischemia;

**(3) MB + ISQ group (*n* = 14):** rats subjected to the CIR protocol and treated with 2 mg/kg methylene blue (MB) i.v. through the left femoral vein before ischemia;

**(4) ISQ + MB group (*n* = 14):** rats subjected to the CIR protocol and treated with 2 mg/kg MB i.v. through the left femoral vein after ischemia but before reperfusion.

**Figure 1:**
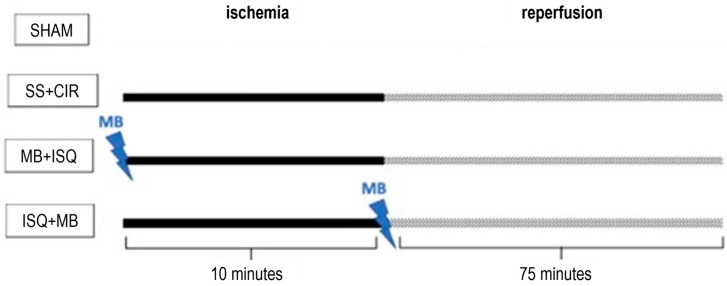
Design of treatment experimental protocols used in the study. In the SHAM-operated group (*n* = 10), rats were subjected to all surgical procedures performed in other groups except for garroting of the left anterior descending coronary artery. In the SS + CIR group (*n* = 14), rats were subjected to CIR protocol and treated with i.v. 0.9% saline solution (SS) administered before ischemia. In the MB + ISQ group (*n* = 14), rats were subjected to the CIR protocol and treated with i.v. 2 mg/kg methylene blue (MB) before cardiac ischemia. In the ISQ + MB group (*n* = 14), rats were subjected to the CIR protocol and treated with i.v. 2 mg/kg MB after cardiac ischemia but before reperfusion.

Figure 1 shows the experimental protocol designs for the treatments used in the present study:

### 2.3. Biochemical Analusis of Serum Biomarkers of Cardiac Injury

At the end of the CIR protocol, blood samples were drawn from the abdominal aorta and centrifuged for 40 min at 2500 rpm, 5 °C. To prepare it for biochemical examination, the serum was kept at −20 °C. Quantitative determination of creatine kinase (CK) and creatine kinase MB fraction (CK-MB) was carried out using a kinetic UV technique with a measurement point of 340 nm with a commercial kit (Katal Biotecnológica Ind. Com. Ltd.a., Belo Horizonte, MG, Brazil) [23].

### 2.4. Biochemical Analysis of Cardiac Lipid Hydroperoxide

In addition, rat cardiac tissue samples were collected to measure LH, homogenized, and centrifuged at 10,000 rpm for 10 min at 20 °C. Subsequently, the supernatant was collected for LH and total protein determination [24]. The quantification of hydroperoxide concentration was performed according to the technique described by [25], considered one of the most sensitive methods for determining lipid peroxidation. The lipid oxidation process occurs due to the release of hydrogen and the addition of molecular oxygen and, subsequently, the reduction of hydroperoxyl radicals to hydroperoxides. The principle of the technique used in the present study consists, briefly, of the oxidation of ferrous ions (Fe^2+^) to ferric ions (Fe^3+^) by hydroperoxides under acidic conditions. Xylenol orange, a substance used as an indicator, binds to ferric ions, producing a blue-purplish chromophore. The color intensity of this chromophore is directly related to the concentration of lipid hydroperoxide in the sample.

### 2.5. Histopathological Analysis of Left Ventricle Miocardial Tissue

After the surgical protocol for CIR, the abdominal cavity was opened, and the abdominal aorta was accessed and punctured to collect arterial blood. After collecting the blood, the thoracic cavity was opened with an incision along the entire length of the sternum. Then, with sharp scissors, the circulatory vessels were disconnected from the heart, ending with the complete removal of the heart, which was immediately washed with saline solution and placed in a vial with a volume of 20 mL of buffered 10% formalin and sent for analysis by the Histocell pathology laboratory (Sao Paulo, Brazil). Then, the atria and ventricles were separated by a cross-section at the height of the lower atrial edge, and the fragment containing the atria and other adjacent structures was discarded. The fragment containing the left ventricle was again sectioned in axial cross-sections into three parts (apex, mid, and distal regions) of approximately equal thickness, which were subsequently dehydrated in progressive concentrations of ethanol for 1 h each. The samples were then diaphanized with two 1 h xylol baths, bathed twice for 1 h, and blocked in 60 °C paraffin.

The material was cut into 4–5 µm thick cross-sections, which were washed in 45 °C water and fixed with Mayer’s albumin. After heating for 60 °C for 2 h, tissue sections were deparaffinized and stained with hematoxylin-eosin (HE). The mid-section of the left ventricle myocardium was imaged in the hearts of animals from all groups with a Zeiss Axion Image A2^®^, Oberkochen, Germany, optical microscope (×400 and ×1000) by a skilled pathologist who was blinded to the various groups for descriptive analysis. The presence of hyperemic blood vessels, pyknosis, inflammatory infiltration, cardiomyocyte degeneration, loss of striation, and interstitial edema were evaluated as CIR-related lesions.

### 2.6. Statistical Analysis

Incidences of VA, AVB, and LET were expressed as percentages and compared with Fisher’s exact test. CK-MB and LH levels were expressed as the mean ± standard error of the mean (SEM) and compared with one-way ANOVA with Tukey’s post hoc test. A *p*-value of <0.05 was considered statistically significant. GraphPad Prism 8.0 (GraphPad Software Inc., La Jolla, CA, USA) was used for statistical analysis.

## 3. Results

### 3.1. Incidence of VA, AVB, and LET

**Figure 2:**
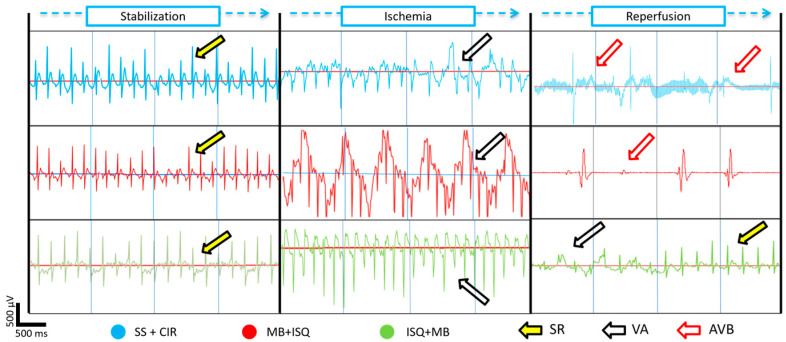
Electrocardiograms of animals belonging to different experimental groups. Representative ECG tracings of rats belonging to the: (Blue color) group treated with saline solution and subjected to the cardiac ischemia and reperfusion protocol (SS + CIR); (Red color) group treated with 2 mg/kg methylene blue (MB) and subjected to cardiac ischemia and reperfusion (MB + ISQ); and (Green color) group treated with 2 mg/kg methylene blue after ischemia but before cardiac reperfusion (ISQ + MB). Yellow arrows indicate sinus rhythm, white arrows indicate ventricular arrhythmias, and red arrows indicate atrioventricular blocks. Representative segments of approximately 4.5 s from each phase are shown.

**Figure 3:**
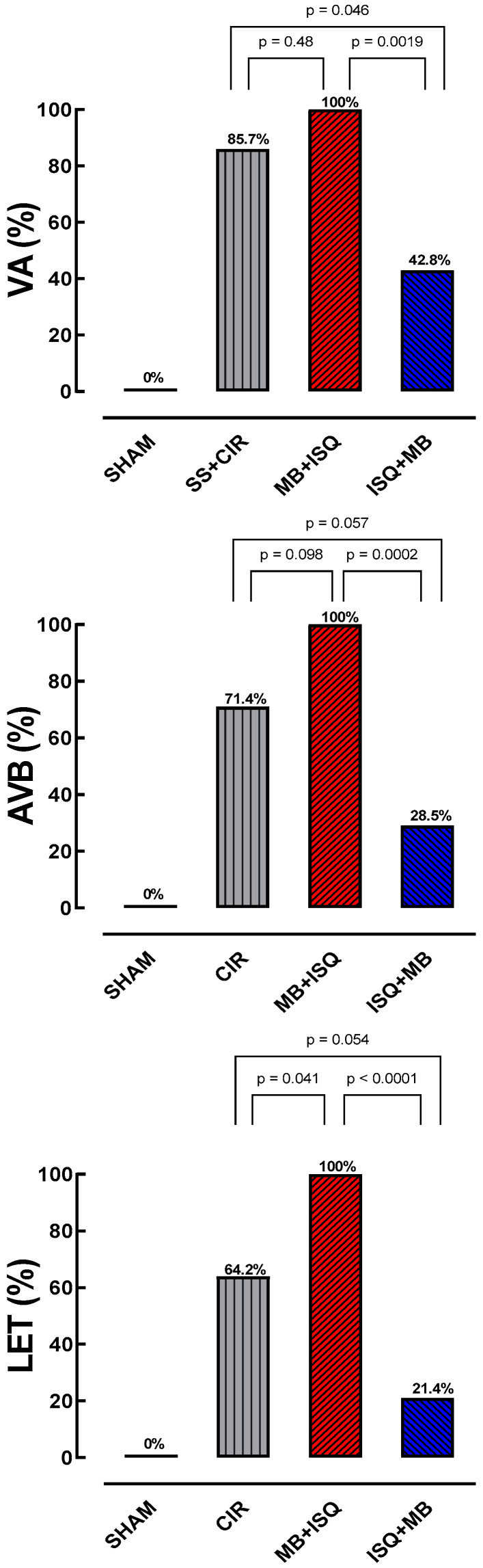
Incidence of ventricular arrhythmias (VAs), atrioventricular block (AVB), and lethality (LET). In the SHAM, SS + CIR, MB + ISQ, and ISQ + MB groups. VA, AVB, and LET increased significantly in the MB + ISQ group when compared with SS + CIR. When methylene blue was administered after ischemia but before reperfusion (ISQ + MB), it significantly reduced VA, AVB, and LET when compared with SS + CIR. SHAM rats had no VA, AVB, or LET. Data were compared using Fisher’s exact test.

Figure 2 and Figure 3 show that administration of MB before ischemia or before reperfusion produced opposite effects on the incidence of VA, AVB, and LET compared with the SS + CIR (control) and SHAM groups. When administered before cardiac ischemia (MB + ISQ), MB significantly increased the incidence of VA (100%), AVB (100%), and LET (100%) compared with SS + CIR (VA = 85.7%; AVB = 71.4%; LET = 64.2%). In contrast, when administered after cardiac ischemia but before reperfusion (ISQ + MB), MB had a significantly protective effect and reduced the incidence of VA (42.8%), AVB (8.5%), and LET (21.4%) compared with SS + CIR.

### 3.2. Histopathological Analysis of the Myocardium

**Figure 4:**
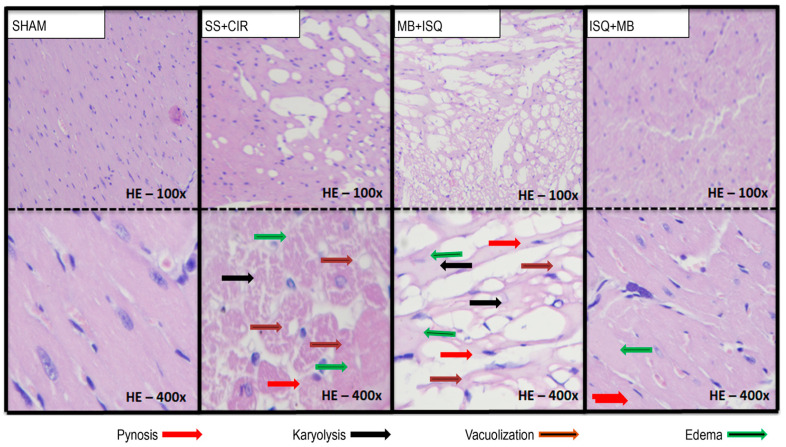
Histopathological analysis of the myocardium of animals belonging to different experimental groups. Representative photomicrographs of histopathological analyses of the rat myocardium from the SHAM, SS + CIR, MB + ISQ, and ISQ + MB groups. In the SHAM group, the myocardium is characterized by the absence of necrosis and striated cells with well-centralized nuclei of normal color and size. In the SS + CIR group, there is intense necrosis due to coagulation, with pyknotic cells (decentralized nuclei and condensation of the chromatin), a marked number of cells undergoing karyolysis (absence of nucleus and cytoplasmic eosinophilia), and swelling and vacuolization. In the MB + ISQ group, similarly, there is intense necrosis due to coagulation, with intense tissue loss, areas of myocytolysis, muscle fibers with swelling (increase in cell volume due to accumulation of water, due to ionic imbalance, reversible), vacuolation, and cells with reduced nuclei or in karyolysis. However, in the MB + ISQ group, the myocardium shows mild necrosis by coagulation, with mild, mild karyolysis, swelling, absence of vacuolization, and preservation of a large part of the striated cells (HE with 400× magnification).

Figure 4 shows that MB administration before ischemia or before reperfusion had opposite histopathological effects on the myocardial tissue when compared with SS + CIR (control). In the SHAM group, the myocardial tissue is free of necrosis, with striated cells with well-centralized nuclei of normal color and size. In the SS + CIR group, the myocardial tissue shows intense necrosis due to coagulation, with pyknotic cells and a marked number of cells undergoing karyolysis and swelling and vacuolization. In the MB + ISQ group, similar findings are observed, with necrosis due to coagulation, intense tissue loss, areas of myocytolysis, muscle fibers with swelling, vacuolation, and cells with reduced nuclei or in karyolysis. However, in the ISQ + MB group, necrosis by coagulation is milder, with mild pyknosis, mild karyolysis, swelling and absence of vacuolization, and preservation of a large part of the striated cells.

### 3.3. Serum Concentration of Total Creatine Kinase (CK) and Creatine Kinase-MB (CK-MB)

**Figure 5:**
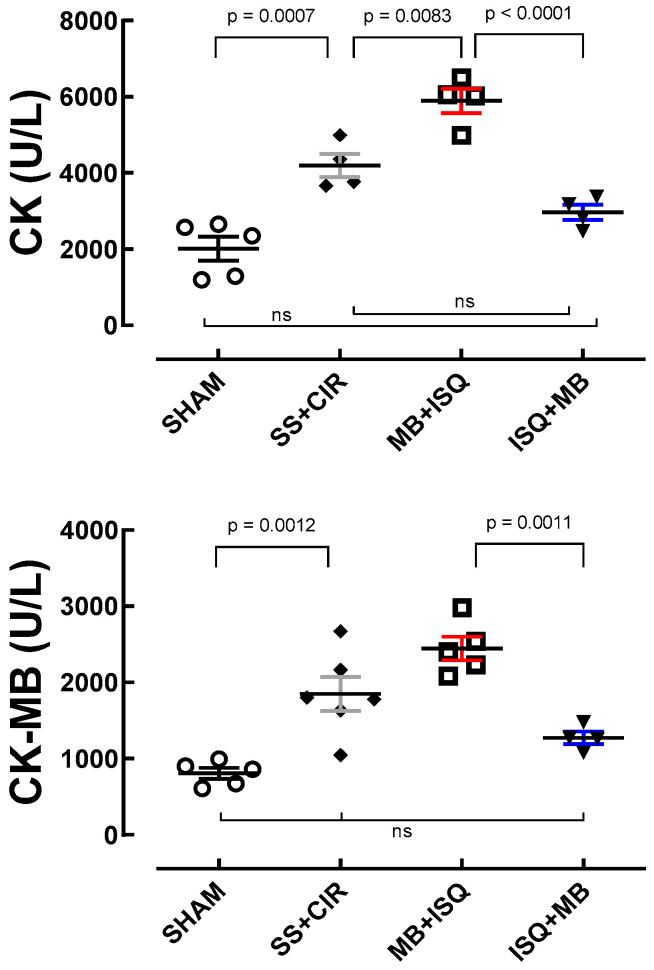
Serum concentrations of biochemical markers of cardiac injury creatine kinase and creatine kinase-MB fraction. Effects of methylene blue (MB) treatment on serum concentrations of total creatine kinase (CK), and creatine kinase-MB (CK-MB) in the SHAM, SS + CIR, MB + ISQ, and ISQ + MB groups. While MB increased CK and CK-MB when administered before ischemia (MB + ISQ), it had an opposite, protective effect when administered after ischemia but before reperfusion (ISQ + MB). Data are shown as scatterplots with the mean ± SEM and were compared with ANOVA followed by Tukey’s post hoc test. ns = represents groups that are not statistically different.

Figure 5 shows that administration of MB before ischemia or before reperfusion also had opposite effects on the serum concentration of biochemical markers of myocardial injury, CK and CK-MB, compared with the SS + CIR and SHAM groups. In the SS + CIR group, the concentrations of CK (4195.7 ± 305.1 U/L, *n* = 4) and CK-MB (1850.8 ± 221.9 U/L, *n* = 4) were higher than the concentrations of CK (2013.4 ± 317.9 U/L, *n* = 4) and CK-MB (807.8 ± 72.1 U/L, *n* = 4) in the SHAM group. When administered before cardiac ischemia (MB + ISQ group), MB significantly increased the concentration of CK (5891.1 ± 320.4 U/L, *n* = 4) and CK-MB (2446.6 ± 154.1 U/L, *n* = 4) compared with the SS + CIR group. In contrast, when administered after ischemia but before cardiac reperfusion (ISQ + MB group), MB significantly reduced the concentration of CK (2013.4 ± 317.9 U/L, *n* = 4) and CK-MB (1274.2 ± 81.9 U/L, *n* = 4), compared with SS + CIR.

### 3.4. Cardiac Concentration of Lipid Hydroperoxide (LH)

**Figure 6:**
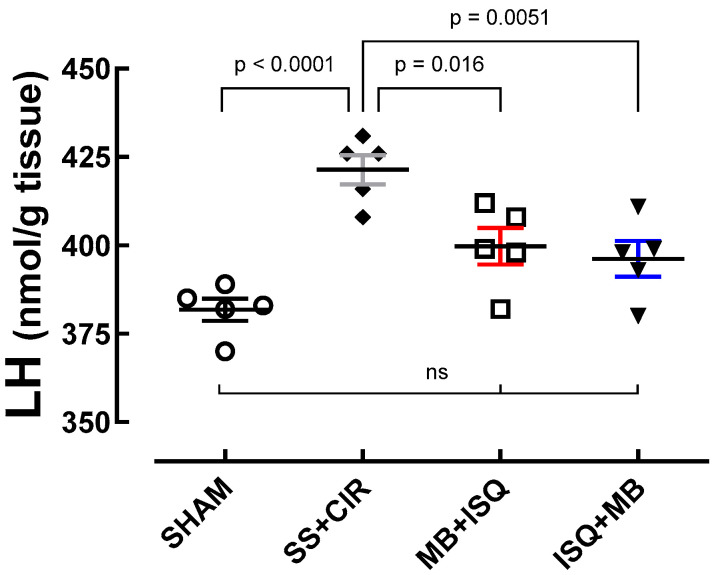
Myocardial concentrations of the biochemical marker of oxidative stress lipid hydroperoxide in the different experimental groups. Cardiac lipid hydroperoxide concentrations in the SHAM, SS + CIR, MB + ISQ, and ISQ + MB groups. Results are expressed as scatterplots with the mean ± SEM (*n* = 5) and were compared with an ANOVA followed by Tukey’s post hoc test. Treatment with MB either before or after ischemia significantly attenuated LH increase seen in the SS + CIR group, though only ISQ + MB LH was reduced to levels not significantly different from the SHAM controls. ns = represents groups that are not statistically different.

Figure 6 shows that the concentrations of LH (nmol/g tissue) in the cardiac tissue of the MB + ISQ, ISQ + MB, and SHAM groups were significantly lower compared with the SS + CIR group. Treatment with MB either before or after ischemia significantly reduced cardiac LH when compared with the SS + CIR group, though only ISQ + MB LH was reduced to levels not significantly different from SHAM controls.

## 4. Discussion

Although MB has been used to treat VS and other conditions related to cardiac surgery in humans [6], the cellular and molecular mechanisms involved in its cardiovascular effects remain under investigation. MB is mostly used due to its inhibition of vascular GC, reducing cGMP synthesis and producing relaxation of the vascular smooth muscle [6,7,8,9,23,24]. Nevertheless, in addition to its important role in vasodilation, cGMP is also involved in cellular responses in other tissues, including in the heart [8]. In cardiac cells, cGMP accounts for the low susceptibility to ventricular fibrillation observed in hearts perfused after sustained ischemia [26,27,28,29,30]. Though MB is supposed to reduce cardioprotective cGMP, the administration of MB in post-cardiac surgery VS and septic shock is associated with the rapid recovery of hemodynamics, lesser need for vasopressors, lower mortality and incidence of renal failure, and shorter length of stay [31,32]. Therefore, it is likely that MB exerts other, yet undescribed, non-cGMP dependent cardioprotective roles.

To promote advancements in the knowledge on these MB effects, the present work studied the effects of MB on cardiac structure and electric activity in rats subjected to CIR. We showed that cardiac effects of MB are highly time-dependent within the ischemia and reperfusion process. The incidence of myocardial injuries, lipid peroxidation, AVB, and LET were significantly increased in the MB + ISQ group compared with the SS + CIR group, suggesting that MB produces cardiotoxic effects when administered early, before ischemia. In contrast, these incidences were significantly lower in the ISQ + MB group, indicating that when ischemia has already taken place, MB has cardioprotective effects when administered before reperfusion. Further investigation about the therapeutic window for the cardiac effects of MB may therefore increase its therapeutic efficacy in cardiac surgery.

The mechanisms through which MB may exert its cardiotoxic or cardioprotective effects remain unclear. In the presence of O_2_ and superoxide radicals, NO chemically reacts to form other products that are extremely cytotoxic and have a high destructive potential for cellular components, such as lipids [10,14], proteins, and nucleic acids, including peroxynitrite (ONOO^−^), in addition to peroxynitrous acid and its decomposition products. (HO^−^ and NO_2_) [33,34]. Indeed, myocardial LH levels were significantly reduced by MB, particularly when it was administered after ischemia. The redox protective effects of MB on mitochondria and inhibition of oxidative stress might be particularly relevant for protection against reperfusion-induced damage [19,35].

In addition to acting as a GC inhibitor, MB acts on L-type Ca^2+^ channels via protein-kinase-G-dependent phosphorylation of Ca^2+^ channels or some associated proteins [36,37,38,39] and binds and blocks M_2_ muscarinic receptors in atrial and ventricular cardiomyocytes [40,41]. Thus, in addition to its protection against structural oxidative damage, MB might directly modulate cardiac electric activity to increase or reduce lethal arrhythmias. The blockage of cardiac M_2_ receptors inhibits vagal negative inotropic and chronotropic effects while accelerating atrioventricular node conduction. The onset of most ventricular tachyarrhythmias occur during ischemia, while lethal AVBs predominate in the later, reperfusion stage. The anti-M_2_ effect of MB might then explain its deleterious role when administered pre-ischemia, when VA and fibrillation constitute the most lethal effects. On the other hand, M_2_ blockage by MB is likely beneficial in preventing lethal AVBs in the reperfusion phase. Nevertheless, MB still aggravated AVB when administered pre-ischemia and attenuated VA post-ischemia. Thus, the biphasic cardiac effect of MB, observed in the present study, appears to be mediated by a combination of multiple actions, such as blockage of cardiac M_2_ receptors, inhibition of NO biosynthesis, modulation of cellular Ca^2+^ homeostasis, mitochondrial bioenergetic [42,43], and oxidation status [44,45,46].

## 5. Conclusions

The experimental findings obtained in the present work indicate that MB treatment could have important implications in cardiac surgery, especially in cardiac transplant. MB treatment can produce cardiotoxic effects when administered before ischemia, representing a risk for cardiac surgery. In contrast, it can also produce cardioprotective effects when administered before reperfusion, representing a new and efficient pharmacological strategy to treat the myocardial injury resulting from reperfusion of the cardiac tissue.

## Data Availability

All data was made available in the publication.

## References

[1] Menezes-Rodrigues F.S., Tavares J.G.P., Vasques E.R., Errante P.R., Araújo E.A., Pires-Oliveira M., Scorza C.A., Scorza F.A., Taha M.O., Caricati-Neto A. (2020). Cardioprotective effects of pharmacological blockade of the mitochondrial calcium uniporter on myocardial ischemia-reperfusion injury. Acta Cir. Bras..

[2] Kumar N., Fitzsimons M.G., Iyer M.H., Essandoh M., Kumar J.E., Dalia A.A., Osho A., Sawyer T.R., Bardia A.J. (2024). Vasoplegic syndrome during heart transplantation: A systematic review and meta-analysis. Heart Lung Transplant..

[3] Mehaffey J.H., Johnston L.E., Hawkins R.B., Charles E.J., Yarboro L., Kern J.A., Ailawadi G., Kron I.L., Ghanta R.K. (2017). Methylene Blue for Vasoplegic Syndrome After Cardiac Operation: Early Administration Improves Survival. Ann. Thorac. Surg..

[4] Gomes W.J., Carvalho A.C., Palma J.H., Gonçalves I., Buffolo E. (1994). Vasoplegic syndrome: A new dilemma. J. Thorac. Cardiovasc. Surg..

[5] Gomes W.J., Carvalho A.C., Palma J.H., Teles C.A., Branco J.N., Silas M.G., Buffolo E. (1998). Vasoplegic syndrome after open heart surgery. J. Cardiovasc. Surg..

[6] Levin R.L., Degrange M.A., Bruno G.F., Del Mazo C.D., Taborda D.J., Griotti J.J., Boullon F.J. (2004). Methylene blue reduces mortality and morbidity in vasoplegic patients after cardiac surgery. Ann. Thorac. Surg..

[7] Evora P.R., Alves Junior L., Ferreira C.A., Menardi A.C., Bassetto S., Rodrigues A.J., Scorzoni Filho A., Vicente W.V.A. (2015). Twenty years of vasoplegic syndrome treatment in heart surgery. Methylene blue revised. Rev. Bras. Cir. Cardiovasc..

[8] de Araújo E.A., Tallo F.S., Oliveira A.S.F., Toghlobi G.S.E., Arantes R.A., Balsimelli R., Kehrwald-Balsimelli B., Bianca Viana B.L.A., Matuda F.S., Nicolau L.A.D. (2024). Cardiotoxic Effects Produced by Omeprazole and Methylene Blue in an Animal Model of Cardiac Ischemia and Reperfusion and Potential Implications for the Pharmacological Strategy for Vasoplegic Syndrome. Biomedicines.

[9] Lenglet S., Mach F., Montecucco F. (2011). Methylene blue: Potential use of an antique molecule in vasoplegic syndrome during cardiac surgery. Expert Rev. Cardiovasc. Ther..

[10] Wolin M.S., Cherry P.D., Rodenburg J.M., Messina E.J., Kaley G.A. (1990). Methylene blue inhibits vasodilation of skeletal muscle arterioles to acetylcholine and nitric oxide via the extracellular generation of superoxide anion. J. Pharmacol. Exp. Ther..

[11] Martin W., Villani G.M., Jothianandan D.E., Furchgott R.F. (1985). Selective blockade of endothelium-dependent and glyceryl trinitrate-induced relaxation by hemoglobin and by methylene blue in the rabbit aorta. J. Pharmacol. Exp. Ther..

[12] Tsai S.C., Adamik R., Manganiello V.C., Vaughan M. (1983). Regulation of activity of purified guanylate cyclase from liver that is unresponsive to nitric oxide. Biochem. J..

[13] Kelner M.J., Bagnell R., Hale B., Alexander N.M. (1988). Potential of methylene blue to block oxygen radical generation in reperfusion injury. Basic Life Sci..

[14] Salaris S.C., Babbs C.F., Voorhees W.D. (1991). Methylene blue as an inhibitor of superoxide generation by xanthine oxidase. A potential new drug for the attenuation of ischemia/reperfusion injury. Biochem. Pharmacol..

[15] Guo R., Tang W., Liu Y. (2024). Protective effect and mechanism of methylene blue on myocardial injury in rats with sépsis. Zhonghua Wei Zhong Bing Ji Jiu Yi Xue.

[16] Tian W.F., Zeng S., Sheng Q., Chen J.L., Weng P., Zhang X.T., Yuan J.J., Pang Q.F., Wang Z.Q. (2018). Methylene blue protects the isolated rat lungs from ischemia-reperfusion injury by attenuating mitochondrial oxidative damage. Lung.

[17] Abreu M.M., Pazetti R., Almeida F.M., Correia A.T., Parra E.R., Silva L.P., Vieira R.P., Pêgo-Fernades P.M., Jatene F.B. (2014). Methylene blue attenuates ischemia-reperfusion injury in lung transplantation. J. Surg. Res..

[18] Semenas E., Nozari A., Sharma H.S., Basu S., Rubertsson S., Wiklund L. (2010). Sex differences in cerebral injury after severe haemorrhage and ventricular fibrillation in pigs. Acta Anaesthesiol. Scand..

[19] Koelzow H., Gedney J.A., Baumann J., Snook N.J., Bellamy M.C. (2002). The effect of methylene blue on the hemodynamic changes during ischemia reperfusion injury in orthotopic liver transplantation. Anesth. Analg..

[20] Tavares J.G.P., Errante P.R., Govato T.C.P., Vasques Ê.R., Ferraz R.R.N., Taha M.O., Menezes-Rodrigues F.S., Caricati-Neto A. (2018). Cardioprotective effect of preconditioning is more efficient than postconditioning in rats submitted to cardiac ischemia and reperfusion. Acta Cir. Bras..

[21] Menezes-Rodrigues F.S., Errante P.R., Ferreira R.M., Tavares J.G.P., Paula L., Araújo E.A., Govato T.C.P., Tikazawa E.H., Reis M.C.M., Luna-Filho B. (2018). Cardioprotective effect of lipstatin derivative orlistat on normotensive rats submitted to cardiac ischemia and reperfusion. Acta Cir. Bras..

[22] Menezes-Rodrigues F.S., Errante P.R., Araújo E.A., Fernandes M.P.P., da Silva M.M., Pires-Oliveira M., Scorza C.A., Scorza F.A., Taha M.O., Caricati-Neto A. (2021). Cardioprotection stimulated by resveratrol and grape products prevents lethal cardiac arrhythmias in an animal model of ischemia and reperfusion. Acta Cir. Bras..

[23] Menezes-Rodrigues F.S., Errante P.R., Tavares J.G.P., Ferraz R.R.N., Gomes J.G., Taha M.O., Scorza C.A., Scorza F.A., Caricati-Neto A. (2019). Pharmacological modulation of β-adrenoceptors as a new strategy for therapy of myocardial dysfunction induced by ischemia and reperfusion. Acta Cir. Bras..

[24] Jiang Z.Y., Woollard A.C., Wolff S.P. (1991). Lipid hydroperoxide measurement by oxidation of Fe2+ in the presence of xylenol orange. Comparison with the TBA assay and an iodometric method. Lipids.

[25] Nourooz-Zadeh J., Rahimi A., Tajaddini-Sarmadi J., Tritschler H., Rosen P., Halliwell B., Betteridge D.J. (1997). Relationships between plasma measures of oxidative stress and metabolic control in NIDDM. Diabetologia.

[26] Ortoleva J., Dalia A.A., Pisano D.V., Shapeton A. (2024). Diagnosis and Management of Vasoplegia in Temporary Mechanical Circulatory Support: A Narrative Review. J. Cardiothorac. Vasc. Anesth..

[27] Evora P.R. (2016). Methylene blue is a guanylate cyclase inhibitor that does not interfere with nitric oxide synthesis. Tex. Heart Inst. J..

[28] Miki N., Kawabe Y., Kuriyama K. (1977). Activation of cerebral guanylate cyclase by nitric oxide. Biochem. Biophys. Res. Commun..

[29] Evora P.R.B., Soares R.O.S., Bassetto S., Martins M.A., Silva F.L.D.S., Basile A.F. (2021). After Thirty Years, We Still Cannot Understand Why Methylene Blue Is Not a Reference to Treat Vasoplegic Syndrome in Cardiac Surgery. Braz. J. Cardiovasc. Surg..

[30] Pabla R., Bland-Ward P., Moore P.K., Curtis M.J. (1995). An endogenous protectant effect of cardiac cyclic GMP against reperfusion-induced ventricular fibrillation in the rat heart. Br. J. Pharmacol..

[31] McCartney S.L., Duce L., Ghadimi K. (2018). Intraoperative vasoplegia: Methylene blue to the rescue!. Curr. Opin. Anaesthesiol..

[32] Habib A.M., Elsherbeny A.G., Almehizia R.A. (2018). Methylene blue for vasoplegic syndrome postcardiac surgery. Indian J. Crit. Care Med..

[33] Huang C., Cui Y., Ji L., Zhang W., Li R., Ma L., Xing W., Zhou H., Chen B., Yu J. (2013). Catalpol decreases peroxynitrite formation and consequently exerts cardioprotective effects against ischemia/reperfusion insult. Pharm. Biol..

[34] Hossne N.A., Miranda M., Monteiro M.R., Branco J.N., Vargas G.F., Pestana J.O., Gomes W.J. (2015). Cardiopulmonary bypass increases the risk of vasoplegic syndrome after coronary artery bypass grafting in patients with dialysis-dependent chronic renal failure. Rev. Bras. Cir. Cardiovasc..

[35] Micleusc A., Sharma H.S., Martijin C., Wiklund L. (2010). Methylene blue protects the cortical blood-brain barrier against ischemia reperfusion-induced disruptions. Crit. Care Med..

[36] Judenherc-Haouzi A., Zhang X.Q., Sonobe T., Song J., Rannals M.D., Wang J., Tubbs N., Cheung J.Y., Haouzi P. (2016). Methylene blue counteracts H_2_S toxicity-induced cardiac depression by restoring L-type Ca channel activity. Am. J. Physiol. Regul. Integr. Comp. Physiol..

[37] Wang Y.G., Rechenmacher C.E., Lipsius S.L. (1998). Nitric oxide signaling mediates stimulation of L-type Ca^2+^ current elicited by withdrawal of acetylcholine in cat atrial myocytes. J. Gen. Physiol..

[38] Kumar R., Namiki T., Joyner R.W. (1997). Effects of cGMP on L-type calcium current of adult and newborn rabbit ventricular cells. Cardiovasc. Res..

[39] Krejcí A., Michal P., Jakubík J., Rícný J., Dolezal V. (2004). Regulation of signal transduction at M_2_ muscarinic receptor. Physiol. Res..

[40] Abi-Gerges N., Eschenhagen T., Hove-Madsen L., Fischmeister R., Mery P.F. (1997). Methylene blue is a muscarinic antagonist in cardiac myocytes. Mol. Pharmacol..

[41] Yamamoto S., Miyamoto A., Kawana S., Namiki A., Ohshika H. (1998). Role of nitric oxide production through M2-cholinergic receptors in cultured rat ventricular myocytes. Biochem. Biophys. Res. Commun..

[42] Chen J.J., Yu B.P. (1994). Alterations in mitochondrial membrane fluidity by lipid peroxidation products. Free Rad. Biol. Med..

[43] Wen Y., Li W., Poteet E.C., Xie L., Tan C., Yan L.J., Ju X., Liu R., Qian H., Marvin M.A. (2011). Alternative mitochondrial electron transfer as a novel strategy for neuroprotection. J. Biol. Chem..

[44] Cheung J.Y., Wang J., Zhang X.Q., Song J., Tomar D., Madesh M., Judenherc-Haouzi A., Haouzi P. (2018). Methylene blue counteracts cyanide cardiotoxicity: Cellular mechanisms. J. Appl. Physiol..

[45] Ozal E., Kuralay E., Yildirim V., Kilic S., Bolcal C., Kücükarslan N., Gunay C., Demirkilic U., Tatar H. (2005). Preoperative methylene blue administration in patients at high risk for vasoplegic syndrome during cardiac surgery. Ann. Thorac. Surg..

[46] Juffermans N.P., Vervloet M.G., Daemen-Gubbels C.R., Binnekade J.M., de Jong M., Groeneveld A.B. (2010). A dose-finding study of methylene blue to inhibit nitric oxide actions in the hemodynamics of human septic shock. Nitric Oxide.

